# Room-temperature ammonia gas sensing via Au nanoparticle-decorated TiO_2_ nanosheets

**DOI:** 10.1186/s11671-023-03798-5

**Published:** 2023-03-20

**Authors:** Jeong Yun Hwang, Yerin Lee, Gyu Ho Lee, Seung Yong Lee, Hyun-Sik Kim, Sang-il Kim, Hee Jung Park, Sun-Jae Kim, Beom Zoo Lee, Myung Sik Choi, Changhyun Jin, Kyu Hyoung Lee

**Affiliations:** 1 Department of Materials Science and Engineering, Yonsei University, Seoul, 03722 South Korea; 2 KIURI Institute, Yonsei University, Seoul, 03722 South Korea; 3 Department of Materials Science and Engineering, University of Seoul, Seoul, 02504 South Korea; 4 Department of Materials Science and Engineering, Dankook University, Cheonan, 31116 South Korea; 5 Chemland Co., Ltd., Gunpo, 15850 South Korea; 6 Faculty of Nanotechnology and Advanced Materials Engineering, Sejong University, Seoul, 05006 South Korea; 7 School of Nano, Materials Science and Engineering, Kyungpook National University, Sangju, 37224 South Korea

**Keywords:** TiO_2_ nanosheet, Au nanoparticle, Ammonia, Gas sensor, Room temperature

## Abstract

A high-performance gas sensor operating at room temperature is always favourable since it simplifies the device fabrication and lowers the operating power by eliminating a heater. Herein, we fabricated the ammonia (NH_3_) gas sensor by using Au nanoparticle-decorated TiO_2_ nanosheets, which were synthesized via two distinct processes: (1) preparation of monolayer TiO_2_ nanosheets through flux growth and a subsequent chemical exfoliation and (2) decoration of Au nanoparticles on the TiO_2_ nanosheets via hydrothermal method. Based on the morphological, compositional, crystallographic, and surface characteristics of this low-dimensional nano-heterostructured material, its temperature- and concentration-dependent NH_3_ gas-sensing properties were investigated. A high response of ~ 2.8 was obtained at room temperature under 20 ppm NH_3_ gas concentration by decorating Au nanoparticles onto the surface of TiO_2_ nanosheets, which generated oxygen defects and induced spillover effect as well.

**Supplementary Information:**

The online version contains supplementary material available at 10.1186/s11671-023-03798-5.

## Introduction

Serious safety issues like toxic gas leakage, explosion, and fire events frequently burst out due to storage or piping defects in industrial sites dealing with various oxidizing, reducing, and high-pressure hazardous chemical gases [1–9]. Various sensors such as electric capacity-, surface acoustic wave-, catalytic combustion-, optical-, electrochemical-, and semiconductor-type with different operating mechanisms have been developed to prevent these risks in advance [10–15]. Although semiconductor-type sensors have lower selectivity and reliability than electrochemical sensors [16, 17], their simple operation, economically feasible manufacturing process, possible miniaturization, and device compatibility have drawn much attention [18, 19]. The semiconductor-type sensors utilize metal oxides (SnO_2_, ZnO, In_2_O_3_, WO_3_, Ga_2_O_3_, TiO_2_, CuO, TeO_2_, NiO, etc.) as sensing materials [20–22]. Among many metal oxides, TiO_2_ is one of the promising candidates for sensing materials since it has various crystal phases (anatase, rutile, and brookite) [23, 24] and generates carriers [25, 26]. Moreover, multiple forms of TiO_2_ with controlled defect structures can be easily prepared via the sulfuric acid method, chlorine method, chemical vapour deposition (CVD) method, sol–gel method, and anodization method [27, 28]. The easily manipulated defect structures of TiO_2_ are ideal for sensing gas molecules of interest. Different surface atomic sites [29, 30], defects [31], and cross-sectional areas of nanosheets [32] can selectively sense gas molecules with different electron affinities.

Recently, sensitive and selective sensing of ammonia (NH_3_) gas, which is highly corrosive, toxic, yet colourless, has become indispensable along with the increasing use of NH_3_ gas as a non-polluting fuel like hydrogen [33, 34]. Benefitting from easily engineered dimensions and defect structures of TiO_2_, 0- and 1-dimensional TiO_2_-based nanostructures and additional nano-heterostructures were found to be effective in sensing NH_3_ gas with high selectivity, sensitivity, and stability at room temperature [35–40]. Herein, we fabricated the 2-dimensional TiO_2_-based nano-heterostructures to improve NH_3_ gas-sensing performance at room temperature. TiO_2_ nanosheets (NSs) were first synthesized through flux growth and subsequent chemical exfoliation. This is because the flux growth method is advantageous for simultaneously obtaining large single crystals and large monolayer structures [41, 42]. Au nanoparticles (NPs) were then decorated onto the surface of the TiO_2_ NSs by hydrothermal method. The high response of ~ 2.8, twice as high as that of the pristine TiO_2_ NSs, was obtained at an NH_3_ gas concentration of 20 ppm due to the oxygen defects generated in the TiO_2_ NSs as well as the spillover effect activated in the presence of Au NPs [43, 44].

## Experimental procedure

**Fig. 1:**
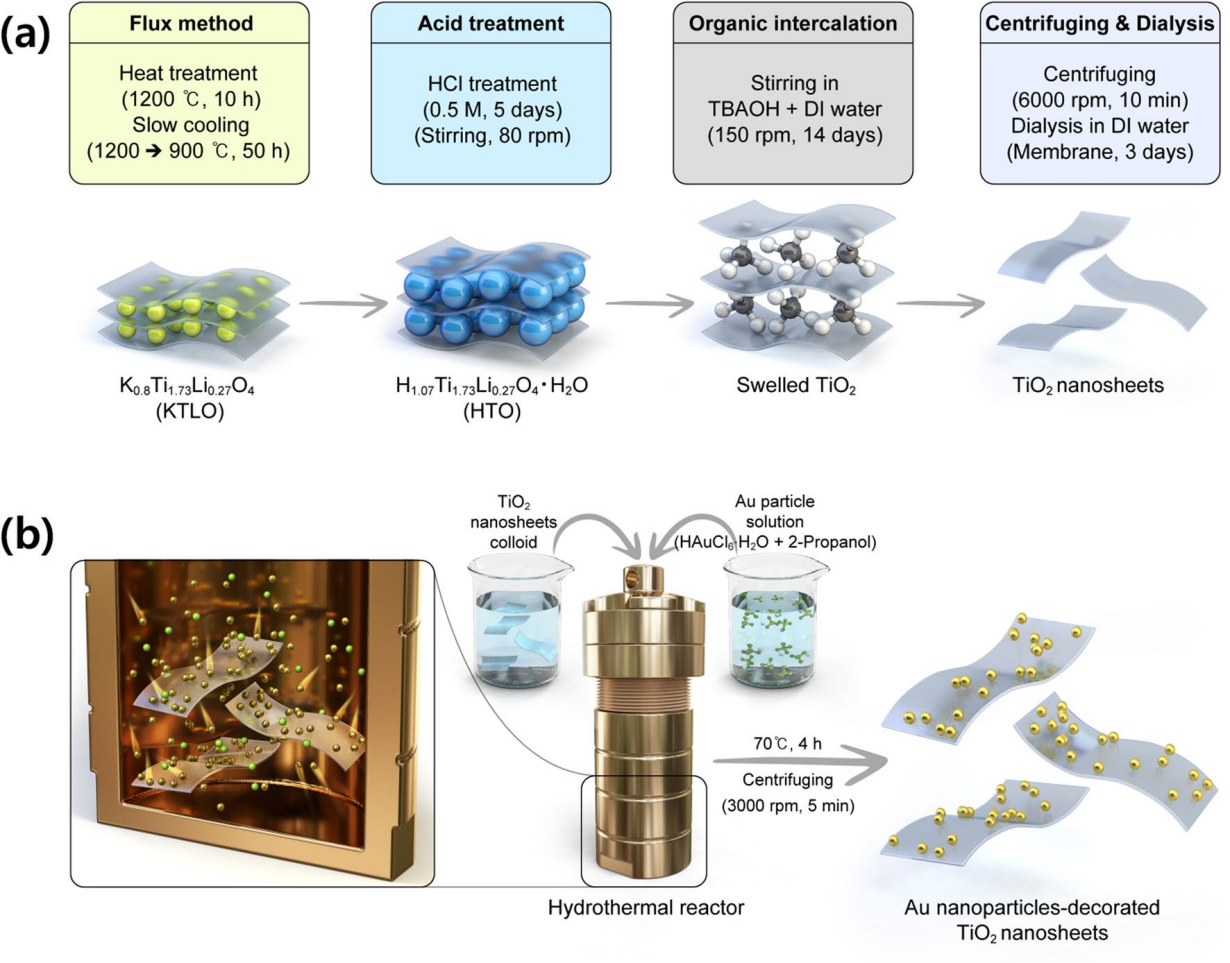
Schematic diagrams for the process of synthesizing **a** TiO_2_ nanosheets via combined process of flux method and exfoliation and **b** Au nanoparticle-decorated TiO_2_ nanosheets via hydrothermal method

As illustrated in Fig. 1a, K0.8Ti_1.73_Li_0.27_O_4_ (KTLO) was first synthesized via a flux method to fabricate TiO_2_ NSs. Powders of TiO_2_ (99.9%, Grand C&M), K_2_CO_3_ (99.5%, Samchun Chemicals), and Li_2_CO_3_ (99.0%, Junsei) were used as starting materials, and MoO_2_ (99.5%, Samchun Chemicals) was added as a flux. Each powder was weighed in stoichiometry and mixed in a mortar. The mixed powder was put into a platinum (Pt) crucible and calcinated at 1200 °C for 10 h in air. After the calcination, it was slowly cooled to 900 °C for 50 h, followed by natural cooling. KTLO powder was obtained once the mixed powder was washed with distilled water at 80 °C to remove K_2_MoO_4_. The KTLO powder was converted to H_1.07_Ti_1.73_O_4_∙H_2_O (HTO) via an acid treatment process performed for 5 days using 0.5 M hydrochloric acid (HCl, Daejung). The HTO powder (1.2 g) was stirred in a mixed solution of tetrabutylammonium hydroxide (TBAOH, 10.26 cc) and distilled (DI) water (300 cc) with a magnetic bar for 14 days to exfoliate TiO_2_ NS. Finally, the TiO_2_ NSs were obtained after centrifuging (6000 rpm, 10 min) and subsequent filtering (membrane in DI water, 3 days) of the TiO_2_ NS-containing mixed solutions.

Au NPs were simply decorated onto the surface of TiO_2_ NSs via the hydrothermal method, as shown in Fig. 1b. The precursor of Au was prepared by stirring HAuCl_6_·H_2_O (0.23 g) and 2-propanol (10 g) for 12 h. The mixture of the Au precursor (HAuCl_6_·H_2_O, 1 ml), colloid of TiO_2_ NSs (4 ml), and distilled water (40 ml) was reacted in an autoclave at 70 °C for 4 h. The Au NP-decorated TiO_2_ NSs were obtained once the product of the hydrothermal reaction was washed in a mixed solution of DI water and ethanol using a centrifuge.

To characterize the morphology, crystallinity, composition, chemical bonding, and surface defect of the pristine and Au NP-decorated TiO_2_ NSs, several analyses using field emission scanning electron microscopy (FE-SEM, JEOL-7001F, Jeol Ltd.), field emission transmission electron microscopy (FE-TEM, Talos F2000X, Thermo Fisher Scientific) with high-angle annular dark field (HAADF), atomic force microscopy (AFM, Park systems), powder X-ray diffraction (PXRD, Smartlab, Rigaku), energy-dispersive X-ray spectroscopy (EDX), X-ray photoelectron spectroscopy (XPS, K-Alpha, Thermo Fisher Scientific), Raman spectroscopy (LabRam Aramis, Horriba Jovin-Yvon), and photoluminescence (PL, LabRam Aramis, Horriba Jovin-Yvon) were carried out.

Sensing properties of the pristine and Au NP-decorated TiO_2_ NSs for various gases (NH_3_, H_2_S, CH_3_COCH_3_, C_6_H_6_, C_2_H_5_OH, NO_2_) were measured using the specially designed gas-sensing measurement system (Supporting Information (SI), Fig. S1), in which sensing materials were deposited on an alumina substrate masked with Au electrode by the drop-off technique. Gas sensing was performed for temperatures ranging from 30 to 110 °C with different gas concentrations (1, 2, 6, 10, and 20 ppm). The response was measured as a change in resistance before (*R*_*a*_) and after (*R*_*g*_) target gas injection. Oxidizing gas and reducing gas were expressed as *R* = *R*_*g*_/*R*_*a*_ and *R* = *R*_*a*_/*R*_*g*_, respectively. Response time and recovery time were calculated as the time to reach 90% of the final resistance in the presence and absence of the target gas, respectively.

## Results and discussion

**Fig. 2:**
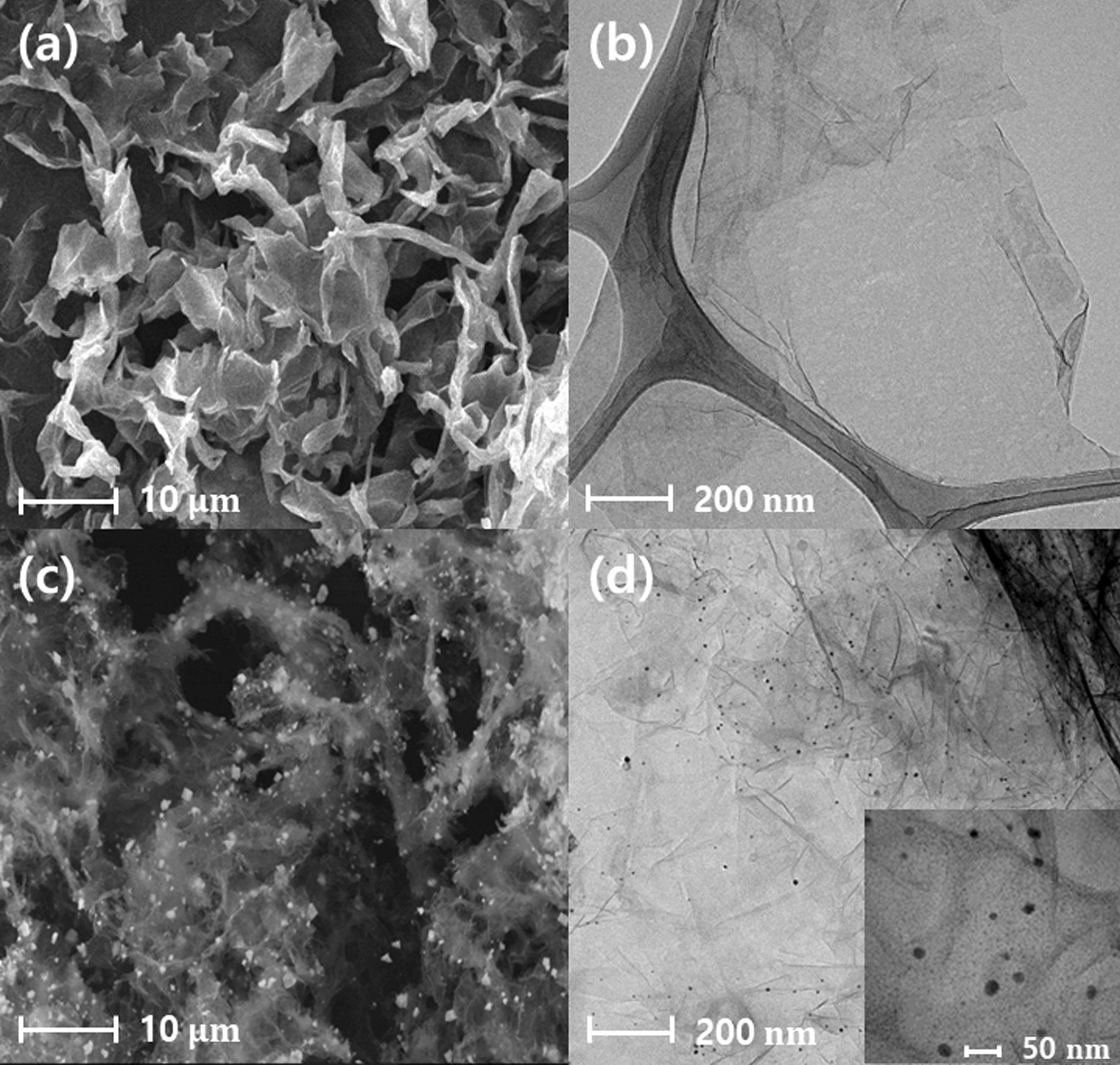
**a** SEM and **b** TEM images showing the morphology of the pristine TiO_2_ nanosheets. **c** SEM and **d** TEM images of the Au nanoparticle-decorated TiO_2_ nanosheets. Inset in (**d**) shows the HADDF image

Figure 2a, b, and SI, Fig. S2a show the SEM and TEM images of the pristine TiO_2_ NSs with uneven and serpentine-like morphology. The TiO_2_ NS thickness of ~ 1.4 nm estimated from the AFM image (SI, Fig. S2b) indicates a successful insertion of TBA^+^ ion between monolayer TiO_2_ sheets just before the exfoliation (Fig. 1a), because the thickness of monolayer TiO_2_ NS (~ 0.5 nm) and the size of adsorbed TBA^+^ ion are approximately 0.5 and 0.9 nm, respectively [45]. Monodispersed Au NPs with an average diameter of 10–30 nm were decorated onto the surface of TiO_2_ NSs via the simple and scalable hydrothermal process (Fig. 1b), as shown in Fig. 2c, d. The decorated Au NPs were not detached from TiO_2_ NSs even after ultrasonication for 24 h.

**Fig. 3:**
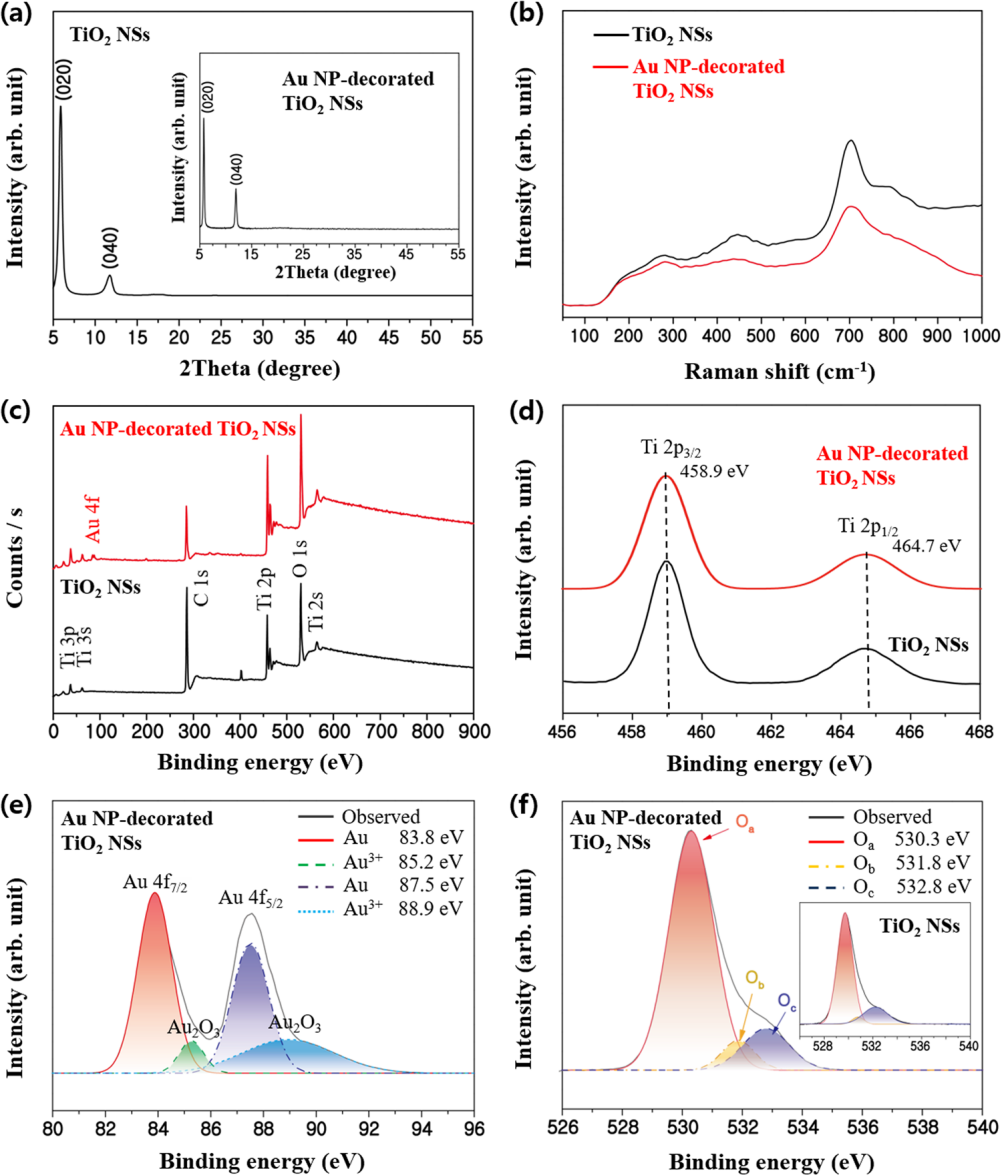
**a** XRD patterns, **b** Raman spectra, and **c**–**f** XPS surveys of the pristine and Au nanoparticle-decorated TiO_2_ nanosheets

Crystal structure, chemical bonding, and defect structure of the pristine and Au NP-decorated TiO_2_ NSs were examined using XRD, Raman, PL, and XPS since the gas-sensing properties of semiconducting metal oxides are strongly correlated with these structural characteristics. Observed peaks in XRD analysis (Fig. 3a) are well matched to the planes of (0*l*0), which confirms the 2-dimensional sheet-like structure of TiO_2_ NSs [46]. This 2-dimensional feature is maintained even after the hydrothermal process for Au NPs decoration. Peaks for Au are not detected, suggesting that the Au NPs with uniform nm-scale size are decorated as confirmed in the TEM image (Fig. 2d). The presence of such Au NPs can be clearly confirmed through high-resolution transmission electron microscopy (HRTEM) (SI, Fig. S3). However, the HRTEM image showed that the monolayer of TiO_2_ was too thin (~ 0.5 nm); therefore, the interplanar spacing could not be clearly observed. Changes in bonding characteristics and defect structures of TiO_2_ NSs after the decoration of Au NPs are also investigated. In Raman spectra (Fig. 3b), peaks corresponding to the TiO_2_ NS (196, 283, 447, 706, and 784 cm^−1^) are observed both in the pristine and Au NP-decorated TiO_2_ NSs [47], suggesting no significant changes in Ti–O bonding states due to the Au NP decoration. This is also confirmed by XPS analysis, as represented in Fig. 3c, 3d. Peaks for Ti 2 s, Ti 2p, Ti 3 s, Ti 3p, and O 1 s [48] observed in the pristine TiO_2_ NSs are also detected in the Au NP-decorated TiO_2_ NSs at almost the same binding energies (peaks for Ti 2p_3/2_ and Ti 2p_1/2_ at 458.9 and 464.7 eV, respectively in Fig. 3d). The negligible change in the Ti–O bonding after the Au NP decoration can be attributed to the low reaction temperature (70 °C) of the hydrothermal process. On the other hand, the peak of Au 4f is clearly observed in XPS spectra of the Au NP-decorated TiO_2_ NSs (Fig. 3c). Different binding energies for Au (83.8 and 87.5 eV) and Au^3+^ (85.2 and 88.9 eV) are confirmed by the peak analysis (Fig. 3e), indicating the presence of metal Au NPs and the bonding between the Au NPs and TiO_2_. However, defect structures are changed by the Au NP decoration. According to the PL spectra (SI, Fig. S4), the peak intensities of the Au NP-decorated TiO_2_ NSs corresponding to defect structures of oxygen vacancies and Ti interstitials (425 nm and 525 nm) [49, 50] are considerably lower than those measured in the pristine TiO_2_ NSs. The surface oxygen defects, which play a critical role in gas sensing, are generated after the Au NP decoration. As shown in Fig. 3f, O 1s spectra are deconvoluted into three binding states: Ti–O lattice oxygen (O_a_ at ~ 530.3 eV), vacant oxygen (O_b_ at ~ 531.8 eV), and chemisorbed oxygen (O_c_ at ~ 532.8 eV). It is noted that the density of O_b_ was increased from 7.9 to 9.1% after decorating Au NPs. Unlike the Ti–O lattice oxygen binding state, the other two binding states played an important role in accelerating the properties of n-type semiconductors. When the vacancies of oxygen (O_b_ in Fig. 3f) increased, the ratio of fixed charge Ti ions increased. Therefore, the electrons acting as the main carriers of mobile charges also increased. Moreover, with regard to TiO_2_, the electrons acting as the main carriers of mobile charges also increased because the increase in chemisorbed oxygen (Au_2_O_3_ in Fig. 3e and O_c_ in Fig. 3f) provided the same effect as that provided by disappearing O. In general, a heterointerface occurring in a pristine metal oxide nanostructure is commonly accompanied by a carrier transfer [51]. However, in our study, when Au was decorated onto TiO_2_ NSs, the overall Ti frame was maintained according to the Raman (Fig. 3b) and XPS (Fig. 3d) spectra. Meanwhile, O was considered to be locally chemisorbed with Au (Au_2_O_3_ in Fig. 3e and O_c_ in Fig. 3f) or to have acted as a vacancy defect (O_b_ in Fig. 3f).

**Fig. 4:**
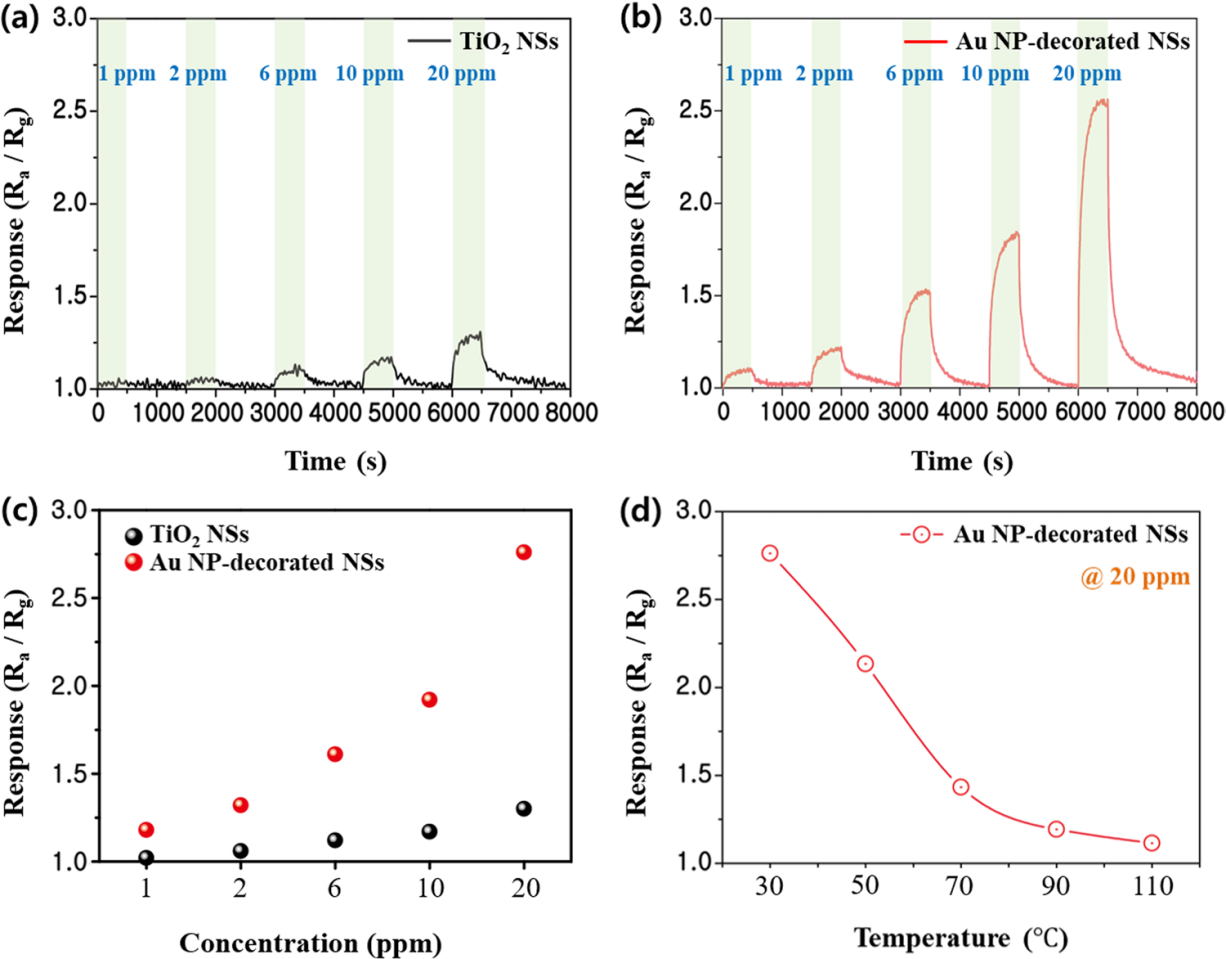
NH_3_ gas concentration-dependent responses at room temperature of **a** the pristine TiO_2_ nanosheets and **b** Au nanoparticle-decorated TiO_2_ nanosheets. **c** Comparison of the NH_3_ gas concentration-dependent responses at room temperature between the pristine and Au nanoparticle-decorated TiO_2_ nanosheets. **d** Response of the Au nanoparticle-decorated TiO_2_ nanosheets according to the process temperature under 20 ppm NH_3_ gas concentration

To investigate the reactivity of the pristine and Au NP-decorated TiO_2_ NSs with NH_3_ gas correlated to the overall characteristics of morphology, chemical bonding, and surface defects characterized as above, their responses under NH_3_ gas concentrations of 1–20 ppm at room temperature were measured (Fig. 4a, b). Owing to the n-type semiconducting nature of TiO_2_, its resistance decreased when TiO_2_ chemically reacted with the reducing NH_3_ gas. In the initial state, the reducing NH_3_ gas supplied electrons to the TiO_2_ semiconductor. The response of the Au NP-decorated TiO_2_ NSs is significantly improved in reference to that of the pristine TiO_2_ NSs, even at a low concentration of 1 ppm. Moreover, considering the high sensing process temperatures (200–300 °C) of most semiconducting metal oxides [52, 53], sensitive sensing demonstrated at room temperature suggests that this low-dimensional nano-heterostructured material is a promising candidate for NH_3_ gas-sensing materials. Dynamic responses for varying NH_3_ gas concentrations are shown in Table S1. In both samples, the response gradually decreased when the concentration of NH_3_ gas increased. The high response of ~ 2.8 is achieved with Au NP-decorated TiO_2_ NSs at 20 ppm NH_3_ gas concentration, which is approximately 6 times higher than that obtained with the pristine TiO_2_ NSs (Fig. 4c). The ionization of oxygen and the adsorption of NH_3_ gas molecules activated by the spillover effect from the decorated Au NPs are the physics behind the improved response [54]. Conventionally, the response of a semiconductor increases with temperature as more carriers are generated at high temperatures. In contrast, the opposite trend is observed in the response of the Au NP-decorated TiO_2_ NSs shown in Fig. 4d. This phenomenon can be attributed to the thin 2D structure of the sample used herein. When the temperature increased, the sample exhibited the properties of a metal rather than those of a semiconductor. Therefore, the number of carriers involved in sensing decreased with temperature. Room-temperature sensing can be understood with the following ionization processes, which occurs below 100 °C [55].

1 $${\text{O}}_{{{2}({\text{gas}})}} \leftrightarrow {\text{ O}}_{{{2}({\text{ads}})}}$$

2 $${\text{O}}_{{\text{2(ads)}}} + \, e^{ - } \to {\text{ O}}_{{\text{2(ads)}}}^{ - }$$

Additionally, Au NP-decorated TiO_2_ NSs can selectively sense NH_3_ gas at room temperature. The responses to other gases such as H_2_S, CH_3_COCH_3_, C_6_H_6_, C_2_H_5_OH, and NO_2_ were 1.41, 1.59, 2.26, 2.06, and 1.43, respectively (SI, Fig. S5). Hence, the selectivity of Au-NP-decorated TiO_2_ NSs in sensing NH_3_ can be considered to be the best. Long-term stability of Au NP-decorated TiO_2_ NSs was confirmed by measurement of sensing dynamics after 1 month, which shows almost same behaviour as shown in SI, Fig. S6. However, humidity performance revealed a slight decrease in response (SI, Fig. S7). Herein, the samples with and without the effect of humidity showed similar responses in the three experiments repeated under the same conditions. Hence, a comparative analysis, as presented in Table 1, was conducted to demonstrate the improved performance of the proposed sensor. [5, 37, 56–61].

**Table 1:** Comparison between the NH_3_ gas-sensing properties of TiO_2_-based gas sensors with the present optimized gas sensor

Sensor	NH_3_ conc (ppm)	Response (R_a_/R_g_)	Temp. (°C)	Refs
TiO_2_ nanotube	1000	~ 2.1	100	[56]
Tanninsulfinic acid-doped polyaniline-TiO_2_ composite	20	1.5	RT	[57]
Cellulose/TiO_2_/PANI composite nanofibers	250	~ 6.5	RT	[58]
TiO_2_ nanowire	50	0.12	RT	[37]
GO/TiO_2_ composite	100	0.383	RT	[5]
PANi-TiO_2_ film	23	1.7	RT	[59]
TiO_2_/PPy	30	3.52	RT	[60]
TiO_2_/SnO_2_ thick film	200	~ 2	250	[61]
Au nanoparticles decorated TiO_2_ nanosheet	20	2.68	RT	Present work

The NH_3_ gas-sensing mechanism for the Au NP-decorated TiO_2_ NSs is closely related to functions of the heterostructures between metal and metal oxide: (1) electronic sensitization with oxygen or adsorbed catalytic metal and (2) chemical sensitization between target gas and catalytic metal or semiconductor surface [62]. For example, the electron affinity of the oxygen adsorbed on the Au-Np-decorated TiO_2_ NS surface is 1.46 eV [63] and that of the TiO with an oxygen vacancy is 1.19 eV [64]. Hence, oxygen removes electrons from the TiO_2_ surface and ionizes the surface as shown in Eq. (2). An electron depletion layer (EDL) is consequently formed under the TiO_2_ surface. Given that Au efficiently decomposes the oxygen (from O_2_ to O_2_^−^) and ammonia (from NH_3_ to N_2_ or NO) adsorbed onto the TiO_2_ surface and helps to form highly reactive ionic species, electron transfer occurs actively. In addition, because Au and TiO_2_ have work functions of 4.86–4.92 eV [65] and 4.34 eV [66], respectively, a Schottky junction [67], in which electrons move only from the TiO_2_ side to the Au side, occurs locally. Therefore, this interface also increases the thickness of the EDL in the air state. However, when NH_3_ gas is injected in this state, the following reaction occurs [68, 69].

3 $$4{\text{NH}}_{{\text{2(ads)}}} + \, 3{\text{O}}_{{\text{2(ads)}}}^{ - } \to \, 2{\text{N}}_{2} + \, 6{\text{H}}_{{2}} {\text{O }} + \, 3e^{ - }$$

4 $$4{\text{NH}}_{{\text{2(ads)}}} + \, 5{\text{O}}_{{\text{2(ads)}}}^{ - } \to \, 4{\text{NO }} + \, 6{\text{H}}_{{2}} {\text{O }} + \, 5e^{ - }$$

Additional electrons are supplied to the surface of TiO_2_ and the thickness of the EDL formed inside the surface is reduced, resulting in a further decrease in resistance. However, when the NH_3_ gas is desorbed, the original EDL thickness is restored owing to the influence of air on the TiO_2_ surface. In summary, the primary gas-sensing mechanism was understood in terms of the conductive channelling width of the semiconductor related to the thickness of the carrier depletion layer [37], where potential difference [70, 71] and energy band diagram [72, 73] models at heterogeneous interfaces (Au NPs and TiO_2_ NSs) could be used.

## Conclusions

Au nanoparticle-decorated TiO_2_ nanosheets are prepared via combination of flux growth-exfoliation and hydrothermal method, and their NH_3_ gas-sensing properties were measured at process temperatures of 30–110 °C under different NH_3_ gas concentrations (1–20 ppm). NH_3_ gas was selectively detected by using the pristine monolayer TiO_2_ nanosheets even at room temperature. The Au nanoparticle decoration triggered the generation of oxygen defects, which activated the reaction with NH_3_ gas molecules, and also induced the spillover effect. Due to this synergetic effect, response of Au nanoparticle-decorated TiO_2_ nanosheets can be significantly improved and reaches ~ 2.8 at room temperature under 20 ppm NH_3_ gas concentration. Proposed design concept of low-dimensional nano-heterostructured sensing material and kinetics of gas sensing at room temperature are expected to be utilized to develop and optimize properties and performances of semiconducting metal oxide-based gas sensors.

## Supplementary Information

Supplementary file 1 (DOCX 2121 kb)

## Data Availability

All the data are available from the corresponding author on reasonable request.
