# Supplementary material for: Room-temperature ammonia gas sensing via Au nanoparticle-decorated TiO2 nanosheets
Source: Discov Nano. 2023 Mar 20;18(1):47. doi: 10.1186/s11671-023-03798-5 (PMC10214923; doi:10.1186/s11671-023-03798-5)
Supplement: Supplementary file 1 — (DOCX 2121 kb) [file 11671_2023_3798_MOESM1_ESM.docx]

**Supporting Information**

**Room temperature ammonia gas sensing via Au nanoparticle-decorated TiO_2_ nanosheets**

Jeong Yun Hwang^a,1^, Yerin Lee^a,1^, Gyu Ho Lee^a^, Seung Yong Lee^a,b^, Hyun-Sik Kim^c^, Sang-il Kim^c^, Hee Jung Park^d^, Sun-Jae Kim^e^, Beom Zoo Lee^f^, Myung Sik Choi^g,*^, Changhyun Jin^a,*^, and Kyu Hyoung Lee^a,*^

^a^ Department of Materials Science and Engineering, Yonsei University, Seoul 03722, South Korea

^b^ KIURI Institute, Yonsei University, Seoul 03722, South Korea

^c^ Department of Materials Science and Engineering, University of Seoul, Seoul 02504, South Korea

^d^ Department of Materials Science and Engineering, Dankook University, Cheonan 31116, South Korea

^e^ Chemland Co., Ltd., Gunpo 15850, South Korea

^f^ Faculty of Nanotechnology and Advanced Materials Engineering, Sejong University, Seoul 05006, South Korea

^g^ School of Nano & Materials Science and Engineering, Kyungpook National University, Sangju 37224, South Korea

^1^ Jeong Yun Hwang and Yerin Lee had equal contribution as co-first authors.

*Correspondence to: ms.choi@knu.ac.kr (M.S.C.), z8015026@yonsei.ac.kr (C.J.), khlee2018@yonsei.ac.kr (K.H.L.)


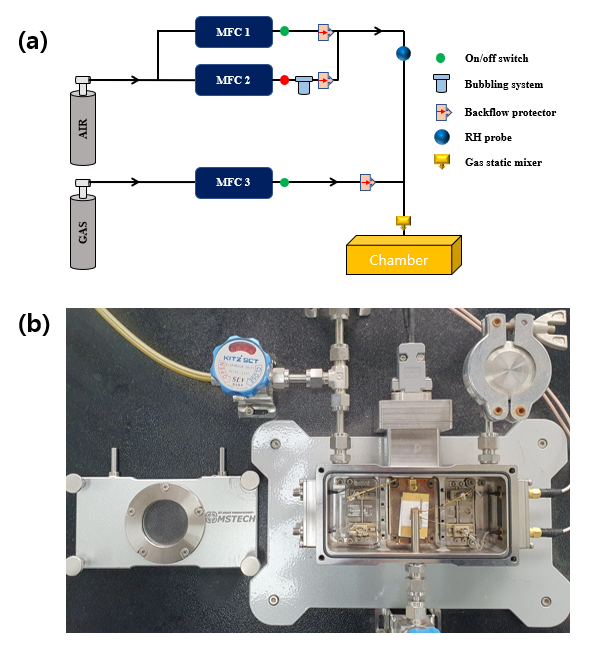


Figure S1. (a) A simple schematic diagram showing the principle of operation in a gas sensor system, (b) Actual image of the gas sensor system.


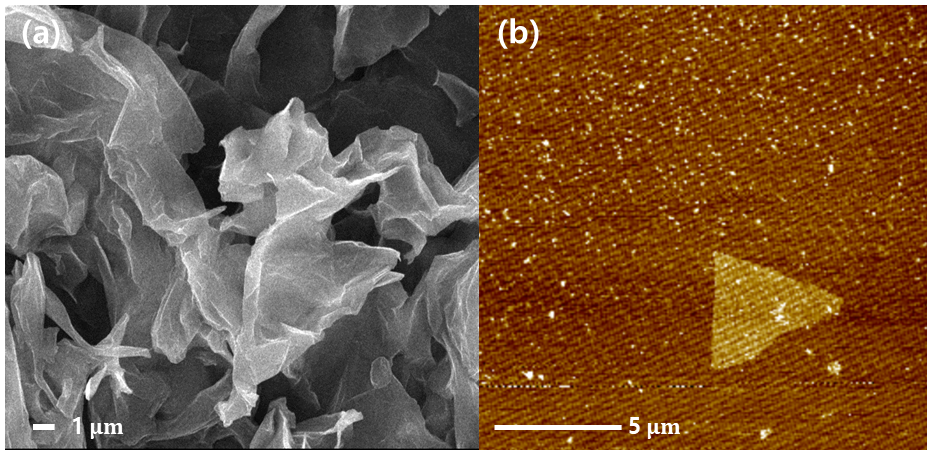


Figure S2. (a) SEM and (b) AFM images of the pristine TiO_2_ nanosheets.


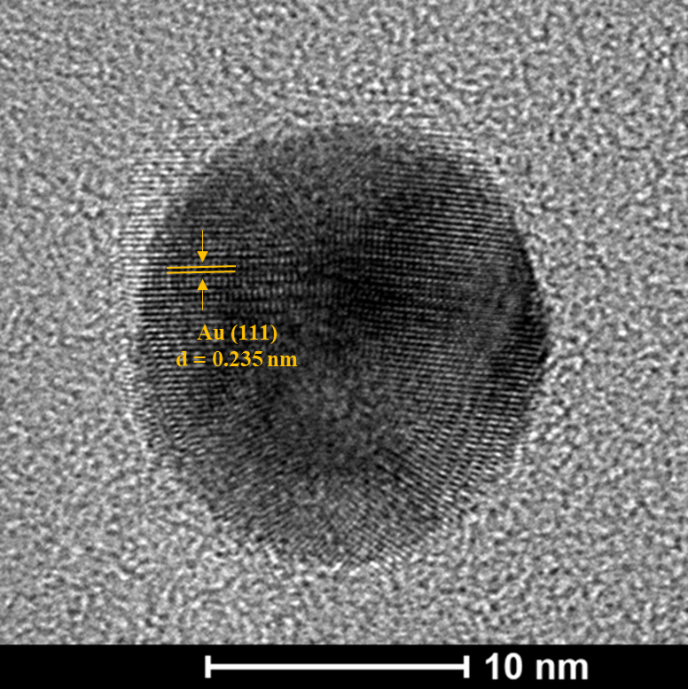


Figure S3. HRTEM image of Au nanoparticle-decorated TiO_2_ nanosheets.


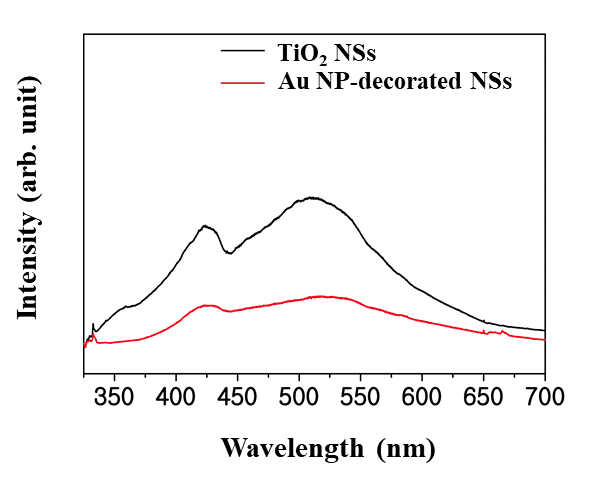


Figure S4. PL spectra of the pristine and Au nanoparticle-decorated TiO_2_ nanosheets.


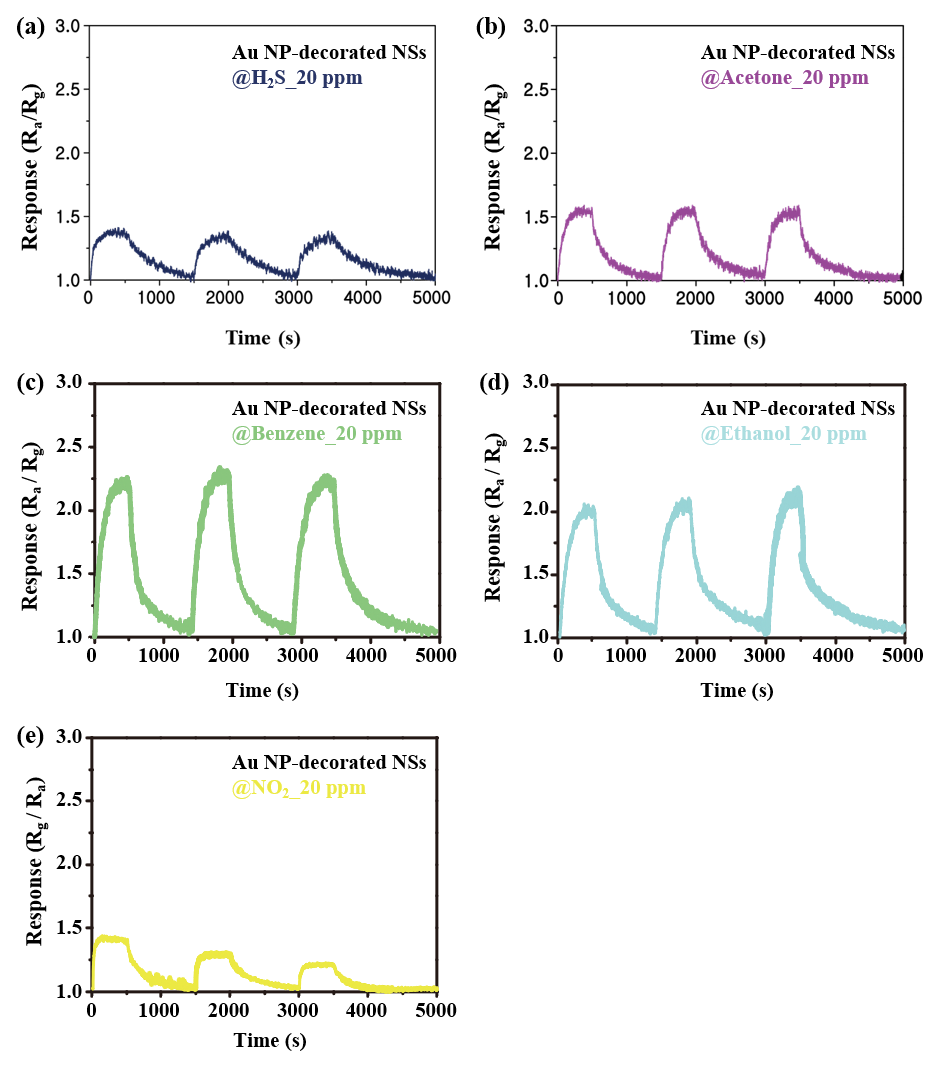


Figure S5. Responses of the Au nanoparticle-decorated TiO_2_ nanosheets for (a) H_2_S, (b) CH_3_COCH_3_, (c) C_6_H_6_, (d) C_2_H_5_OH, and (e) NO_2_ gas under 20 ppm concentration.


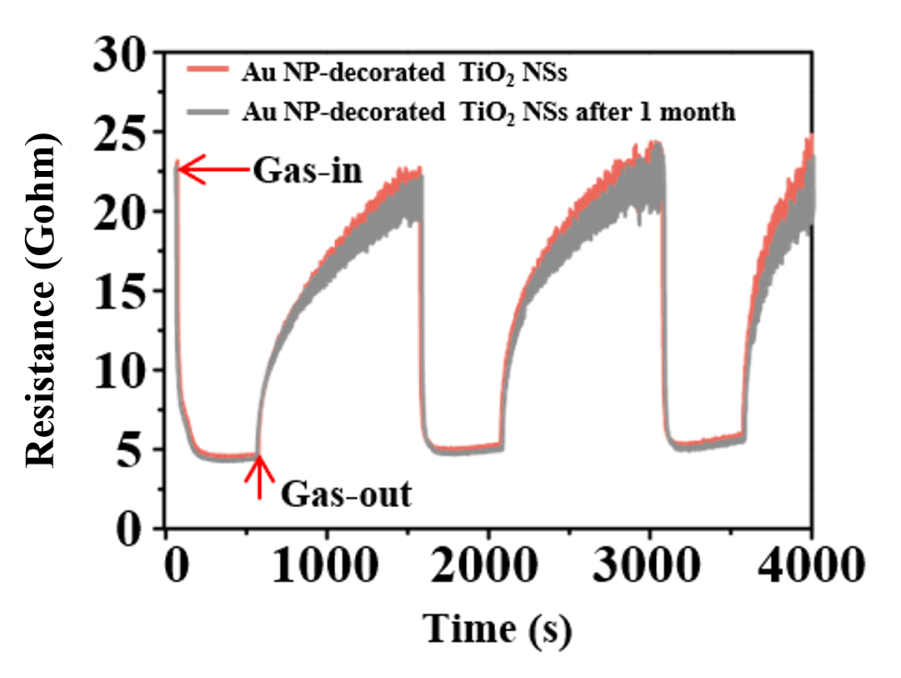


Figure S6. Dynamic resistance curves of the Au nanoparticle-decorated TiO_2_ nanosheets under 20 ppm NH_3_ gas in the fresh state and after keeping in the laboratory for 1 month.


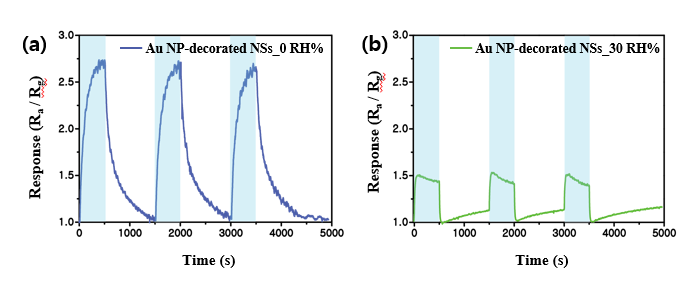


Figure S7. Responses of the Au nanoparticle-decorated TiO_2_ nanosheets under 20 ppm NH_3_ gas in the presence of (a) 0% and (b) 30% relative humidity.

Table S1. Dynamic responses according to NH_3_ gas concentration.

|  | TiO_2_ nanosheets  (R_a_ / R_g_) | Au nanoparticle-decorated TiO_2_ nanosheets  (R_a_ / R_g_) |
| --- | --- | --- |
| 1 ppm | 1.02 | 1.18 |
| 2 ppm | 1.06 | 1.32 |
| 6 ppm | 1.12 | 1.61 |
| 10 ppm | 1.17 | 1.92 |
| 20 ppm | 1.30 | 2.76 |
